# Enhancing the performance of the vehicle active suspension system by an Optimal Sliding Mode Control algorithm

**DOI:** 10.1371/journal.pone.0278387

**Published:** 2022-12-01

**Authors:** Duc Ngoc Nguyen, Tuan Anh Nguyen

**Affiliations:** Automotive Engineering Department, Thuyloi University, Hanoi, Vietnam; Federal University of Technology - Parana, BRAZIL

## Abstract

The suspension system determines riding comfort. This item utilizes an active suspension system to absorb vehicle vibration. A quarter-dynamics model with five state variables simulates the oscillations of a vehicle. This model incorporates the hydraulic actuator effect into linear differential equations. This is an entirely original design. In addition, the OSMC (Optimal Sliding Mode Control) algorithm is proposed for active suspension system operation control. The in-loop algorithm optimizes the controller’s parameters. According to the findings of the study, when the OSMC algorithm was implemented, the maximum and average displacement values of the sprung mass were dramatically lowered under normal oscillation conditions. If a vehicle employs only a passive suspension system or an active suspension system with a standard linear control algorithm, the wheel is fully detached from the road surface in hazardous conditions. When the OSMC algorithm is utilized to control the operation of the active suspension system, the wheel-to-road interaction is always maintained. This algorithm provides a great degree of efficiency.

## 1. Introduction

The suspension system is one of the most important automotive systems. The suspension system has been refined and improved over time. The suspension system ensures that the vehicle’s vibrations do not exceed permissible levels. The passive suspension system is comprised of a coil spring (or leaf spring), a damper, and a lever arm and has a very simple design. In addition, the stabilizer bar is regarded to be a component of the suspension system [1]. The suspension system is situated between the vehicle’s axle and its body. It separates the vehicle into the sprung mass and un-sprung mass [2]. When the vehicle’s body vibrates, the internal forces generated by the suspension system control the vibrations by acting on both components of the assembly. The rigidity of these components plays a crucial part in guaranteeing the road-going smoothness of the vehicle. For a passive suspension system, it is impossible to alter the spring or damper stiffness. Therefore, in many instances, smoothness cannot be guaranteed. Consequently, a number of automatically controlled suspension systems have replaced conventional mechanical suspensions. In [3], Zepeng et al. introduced car air suspension. This suspension system replaces the metal spring with an air balloon (air spring). As air is added to or removed from a balloon, the internal pressure fluctuates. This causes the spring stiffness to vary continuously in response to the driving conditions of the vehicle [4]. Changing the damper’s stiffness is an additional concern. Khedkar et al. provided a model of electronic damping with an average response in [5]. The arrangement of iron particles within the damper determines the pace of liquid circulation. When the body of the car vibrates, an electric current signal is applied to the damper’s inside. The appearance of a magnetic field will alter the arrangement of tiny metal particles. Consequently, the damping stiffness is modifiable [6]. Due to the system’s incomplete responsiveness, the suspension system is referred to as semi-active. Ultimately, an active suspension system was utilized to enhance the suspension system. As part of the dynamic suspension, every wheel includes a hydraulic actuator. The operation of this mechanism depends on the opening and closing of servo valves. Controlling the opening and closing of the valve gates is the controller’s current signal. According to Nguyen, the hydraulic actuator will exert force on the spring-loaded and un-sprung masses. This force is utilized to dampen the vehicle’s vibrations. From there, the vehicle’s smoothness will be substantially enhanced [7].

Recently, several control algorithms for the active suspension system have been developed. Anh utilized the PID (Proportional–Derivative–Integral) control technique to govern this system in [8]. The PID algorithm is well-suited to SISO (Single Input–Single Output) linear systems. Abdullah et al. presented the MOPID model for active suspension in [9]. This model calls for the employment of three controllers. These three controllers will regulate the system’s K_P_, K_I_, and K_D_ coefficients, respectively. The Ziegler-Nichols approach can be utilized to determine the parameters of the PID controller. However, its precision will be restricted [10, 11]. In addition, sophisticated algorithms, such as GSA (Gravitational Search Algorithm), PSO (Particle Swarm Optimization), etc. [12, 13], allow for a more optimal determination of these parameters. Utilizing a Fuzzy-PID hybrid controller continuously allows for the modification of these parameters’ values. This modification is favourable if the fuzzy rule is constructed properly [14, 15]. The LQR (Linear Quadratic Regulator) control technique is the optimal choice if the system includes many objects to consider, i.e., it is a MIMO (Multi Input–Multi Output) system [16]. According to Maurya and Bhangal’s research, the LQR algorithm employs state matrices rather than the conventional set of differential equations [17]. The LQR algorithm, according to Rodriguez-Guevara et al., is based on the optimization of the cost function [18]. The system’s efficiency can reach a high degree during such time. This technique is frequently paired with a Gaussian filter to reduce the impact of noise [19, 20].

Vehicle oscillations are mostly random in practice. This consists of nonlinear oscillations. In many instances, conventional linear controllers cannot ensure stable performance. Bai and Guo recommended utilizing the SMC (Sliding Mode Control) algorithm for the active suspension system in their paper [21]. For this algorithm, the state variables of the oscillatory state matrix must be determined. If hydraulic actuators are used, there are five (for a quarter-dynamics model) or ten (for a half-dynamics model) potential state variables [22, 23]. This will significantly complicate the process of deriving state variables. Nguyen linearized the hydraulic actuator for the SMC algorithm containing five state variables [24]. The SMC technique can potentially be used with the PSO algorithm to improve controller parameter selection efficiency [25]. Some oscillations are non-stationary and undergo continuous change. The Fuzzy algorithm was therefore proposed to determine these states. The Fuzzy algorithm is applicable to the suspension system, stabilizer bar, and several other control systems [26, 27]. This algorithm can respond to the continuous change of the input signal, as demonstrated by Mustafa et al. in their study [28]. The experience of the designer can be used to establish fuzzy control principles. Moreover, the Fuzzy method is easily combinable with other algorithms [29, 30]. In general, nonlinear and intelligent control approaches are highly efficient [31–34].

The author proposes using the OSMC algorithm for the suspension system in this article. The in-loop algorithm will optimally select the parameters of the Sliding Mode controller. Compared with standard SMC algorithms, the OSMC algorithm can bring higher efficiency. It can be understood that the values of displacement and acceleration of the vehicle body will be reduced more. Besides, the wheel will hold the road better. This can be demonstrated by numerical simulation, which is presented in the sections below.

The main content of this article is divided into four parts, including an introduction section, a methods section, a results section, and a conclusion section. In the first section, some concepts and problems were introduced. In addition, previous studies by other authors have also been clearly analysed and evaluated. The process of setting up the dynamic model and control algorithm will be conducted in the second section. Numerical simulation will be performed in the next section. Moreover, the results that are obtained from the simulation process will also be thoroughly analysed in this section. Finally, some recommendations can be made in the concluding section.

## 2. Methods

### 2.1. Model of the vehicle dynamics

**Fig 1:**
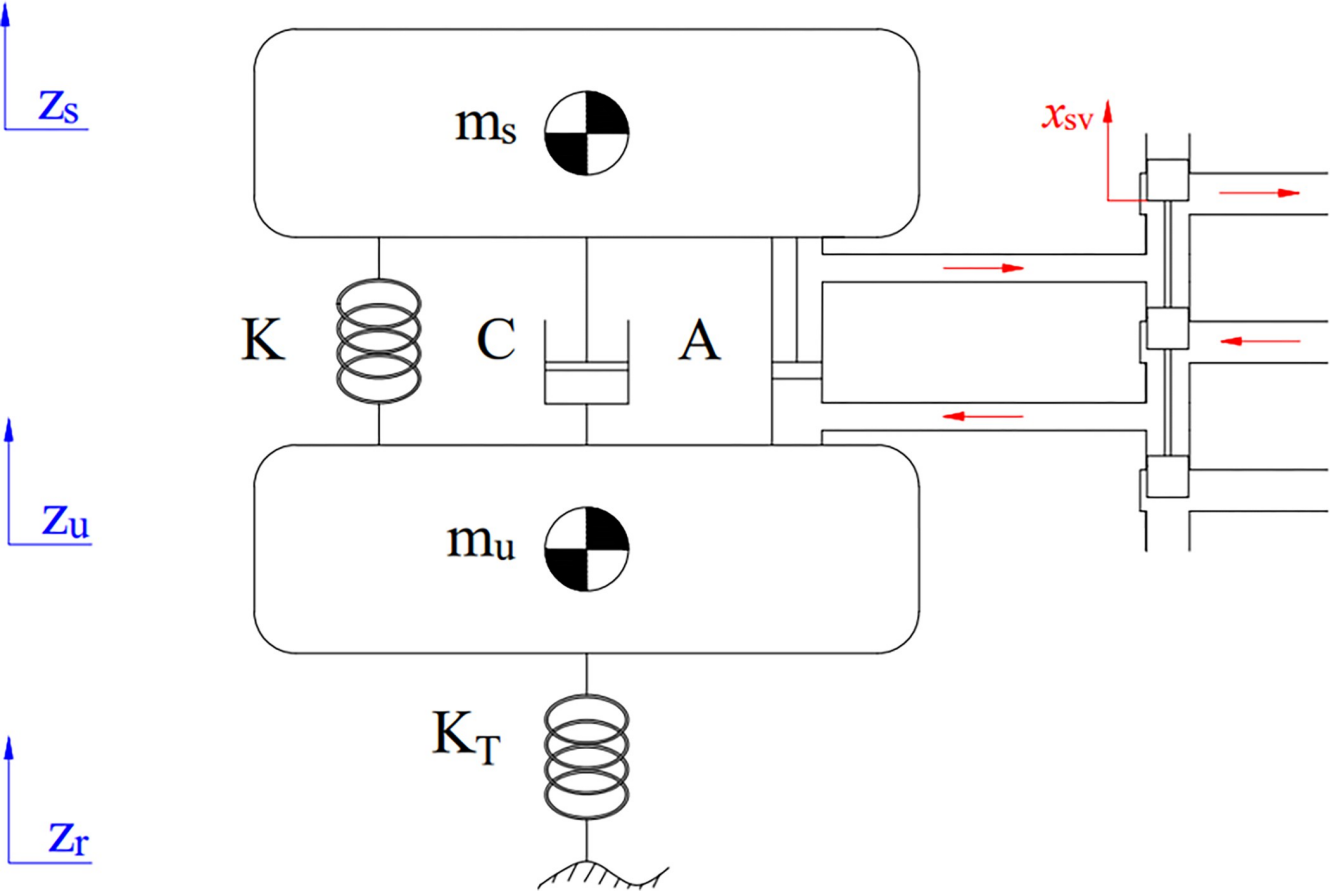
The quarter-dynamics model.

There is a multitude of dynamic models used to represent vehicle vibrations. Frequently, a quarter-dynamics model is applied to control difficulties. This model contains two masses: the spring-loaded mass *m*_*s*_ and the un-sprung mass *m*_*u*_ (Fig 1). This is the form of the system of equations explaining the vibration of the vehicle:

$$F_{is}=F_{K}+F_{C}+F_{A}$$ (1)

$$F_{iu}=F_{KT}-F_{K}-F_{C}-F_{A}$$ (2)

With:

$$F_{is}=m_{s}{\ddot{z}}_{s}$$ (3)

$$F_{iu}=m_{u}{\ddot{z}}_{u}$$ (4)

$$F_{K}=K(z_{u}-z_{s})$$ (5)

$$F_{C}=C({\dot{z}}_{u}-{\dot{z}}_{s})$$ (6)

$$F_{KT}=K_{T}(z_{r}-z_{u})$$ (7)

**Fig 2:**
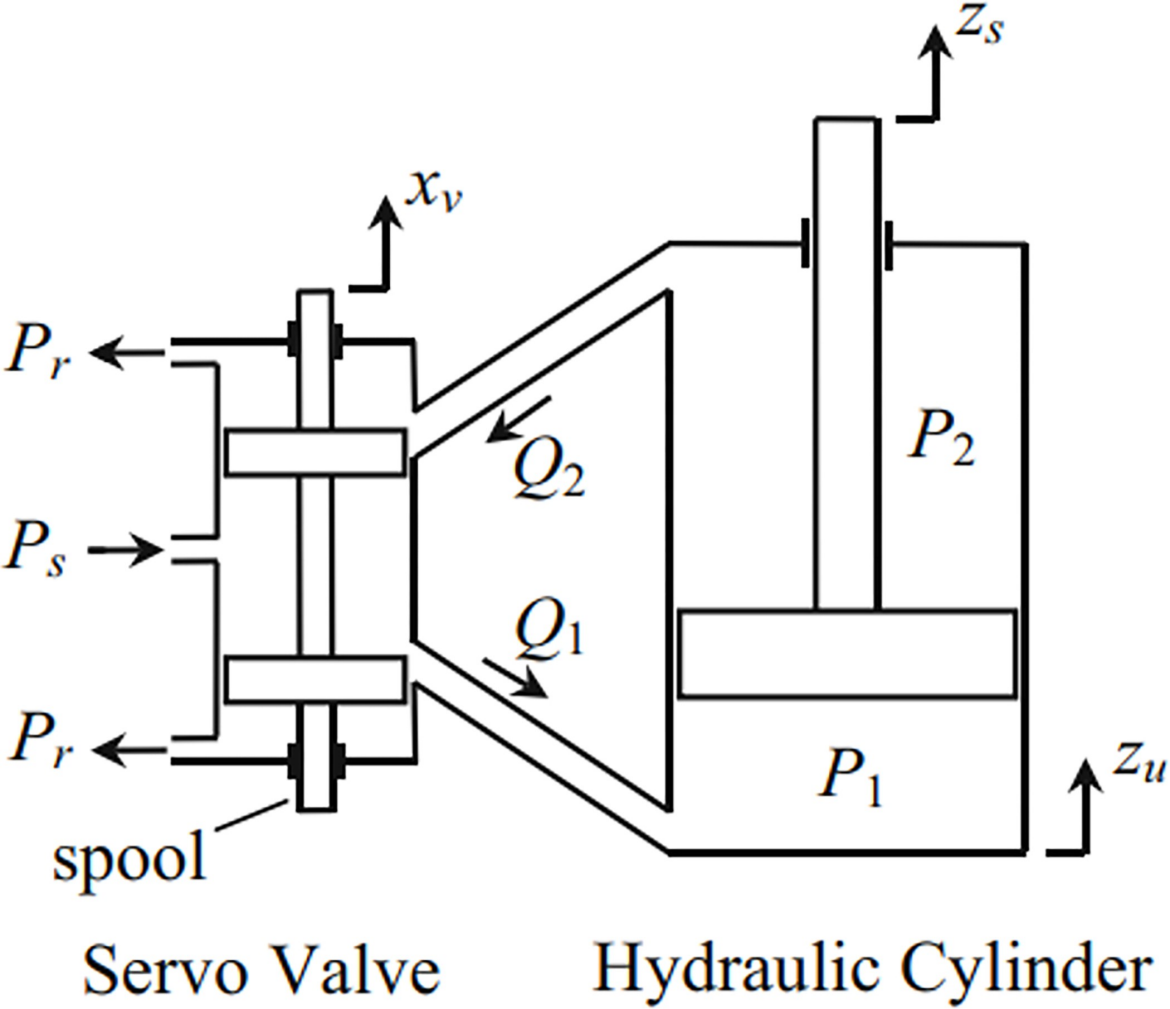
Hydraulic actuator.

The impact force of the actuator (Fig 2) can be determined according to the nonlinear model as follows:

$$F_{a}=AP_{L}$$ (8)

$$P_{L}=P_{1}-P_{2}$$ (9)

$$\frac{V_{t}}{4\beta_{e}}{\dot{P}}_{L}=Q_{L}-C_{t}P_{L}-A({\dot{z}}_{s}-{\dot{z}}_{u})$$ (10)

$$Q_{L}=C_{d}x_{v}w\sqrt{\frac{1}{\rho }[P_{s}-sgn(x_{v})P_{L}]}$$ (11)

Where:

V_t_: effective volume of the hydraulic actuator

β_e_: bulk modulus of the fluid

C_t_: leakage constant

Q_L_: hydraulic load flow

C_d_: discharge coefficient

w: servo valve area gradient

x_v_: servo valve spool displacement

ρ: fluid density

This is a complex dynamic model. For the SMC algorithm, the higher order derivative of the model needs to be used. Therefore, it is necessary to simplify the dynamic model of the actuator. This can make the process of taking the derivative of the state vector easier. Utilizing a linear function simplifies the estimation of the impact force, *F*_*A*_ [24].


$$F_{A}=\kappa_{1}\int (i(t)-\kappa_{2}F_{A})dt+\kappa_{3}z_{sus}$$ (12)

### 2.2. OSMC controller design

The recommended OSMC algorithm is used to control the operation of the active suspension system. Combining Eqs from (1) to (7) and (12), we get:

$$m_{s}{\ddot{z}}_{s}=-Kz_{s}-C{\dot{z}}_{s}+Kz_{u}+C{\dot{z}}_{u}+F_{A}$$ (13)

$$m_{u}{\ddot{z}}_{u}=Kz_{s}+C{\dot{z}}_{s}-(K+K_{T})z_{u}-C{\dot{z}}_{u}-F_{A}+K_{T}z_{r}$$ (14)

For the quarter-dynamics model, the inertia force *F*_*i*_ of the sprung mass and the un-sprung mass is considered to be proportional. This means that:

$${\ddot{z}}_{s}=\chi (z_{r}-z_{u})$$ (15)

Where: *χ* is the proportional factor.

Let:

$$\begin{array}{l} x_{1}=z_{s}\\ x_{2}={\dot{z}}_{s}\\ x_{3}=z_{u}\\ x_{4}={\dot{z}}_{u}\\ x_{5}=F_{A} \end{array}$$ (16)

Taking the derivative of state variables:

$$\begin{array}{l} {\dot{x}}_{1}=x_{2}\\ {\dot{x}}_{2}=\frac{1}{m_{s}}(-Kx_{1}-Cx_{2}+Kx_{3}+Cx_{4}+x_{5})\\ {\dot{x}}_{3}=x_{4}\\ {\dot{x}}_{4}=\frac{1}{m_{u}}(Kx_{1}+Cx_{2}-(K+K_{T})x_{3}-Cx_{4}-x_{5})\\ {\dot{x}}_{5}=-\kappa_{3}x_{2}+\kappa_{3}x_{4}-\kappa_{2}x_{5}+\kappa_{1}i(t) \end{array}$$ (17)

In this article, the value of displacement of the sprung mass is considered as the output of the controller. Therefore:

$$y=x_{1}$$ (18)

Take the *n*-order derivative of the output signal. Where *n* is the number of state variables:

$$\dot{y}=x_{2}$$ (19)

$$\ddot{y}=-\frac{K_{T}}{\chi m_{s}}x_{3}$$ (20)

$$y^{\left(3\right)}=-\frac{K_{T}}{\chi m_{s}}x_{4}$$ (21)

$$y^{\left(4\right)}=-\frac{K_{T}}{\lambda m_{s}m_{u}}(Kx_{1}+Cx_{2}-(K+K_{T})x_{3}-Cx_{4}-x_{5})$$ (22)

$$y^{\left(5\right)}=\frac{K_{T}}{\chi m_{s}m_{u}}(\begin{array}{l} (\frac{KC}{m_{s}}+\frac{KC}{m_{u}})x_{1}+(\frac{C^{2}}{m_{s}}+\frac{C^{2}}{m_{u}}-K-\kappa_{3})x_{2}\\ +(-\frac{KC}{m_{s}}-\frac{(K+K_{T})C}{m_{u}})x_{3}\\ +(-\frac{C^{2}}{m_{s}}-\frac{C^{2}}{m_{u}}+(K+K_{T})+\kappa_{3})x_{4}\\ +(-\frac{C}{m_{s}}-\frac{C}{m_{u}}-\kappa_{2})x_{5} \end{array})+\frac{K_{T}\kappa_{1}}{\chi m_{s}m_{u}}i(t)$$ (23)

Let:

$$\begin{array}{l} b_{1}=\frac{K_{T}}{\chi m_{s}m_{u}}\\ b_{2}=\frac{K_{T}\kappa_{1}}{\chi m_{s}m_{u}}\\ a_{1}=\frac{KC}{m_{s}}+\frac{KC}{m_{u}}\\ a_{2}=\frac{C^{2}}{m_{s}}+\frac{C^{2}}{m_{u}}-K-\kappa_{3}\\ a_{3}=-\frac{KC}{m_{s}}-\frac{(K+K_{T})C}{m_{u}}\\ a_{4}=-\frac{C^{2}}{m_{s}}-\frac{C^{2}}{m_{u}}+(K+K_{T})+\kappa_{3}\\ a_{5}=-\frac{C}{m_{s}}-\frac{C}{m_{u}}-\kappa_{2} \end{array}$$ (24)

Eq (23) can be rewritten as:

$$y^{\left(5\right)}=b_{1}\sum_{i=1}^{5}a_{i}x_{i}+b_{2}i(t)$$ (25)

The sliding surface of the controller is taken according to the error signal with derivative order *n—1*:

$$\sigma =e^{\left(4\right)}+p_{1}e^{\left(3\right)}+p_{2}\ddot{e}+p_{3}\dot{e}+p_{4}e=\sum_{i=0}^{4}p_{i}e^{\left(4-i\right)}$$ (26)

With: *p*_*i*_ being the coefficients of the polynomial.

The output signal of the controller has the form:

$$i(t)=b_{3}(y_{s}^{\left(5\right)}-\sum_{i=1}^{5}a_{i}x_{i}+\sum_{i=1}^{4}p_{i}e^{\left(4-i\right)}+Rsgn(\sum_{i=0}^{4}p_{i}e^{\left(4-i\right)}))$$ (27)

where:

$$b_{3}=\frac{\chi m_{s}m_{u}}{K_{T}\kappa_{1}}$$ (28)

**Fig 3:**
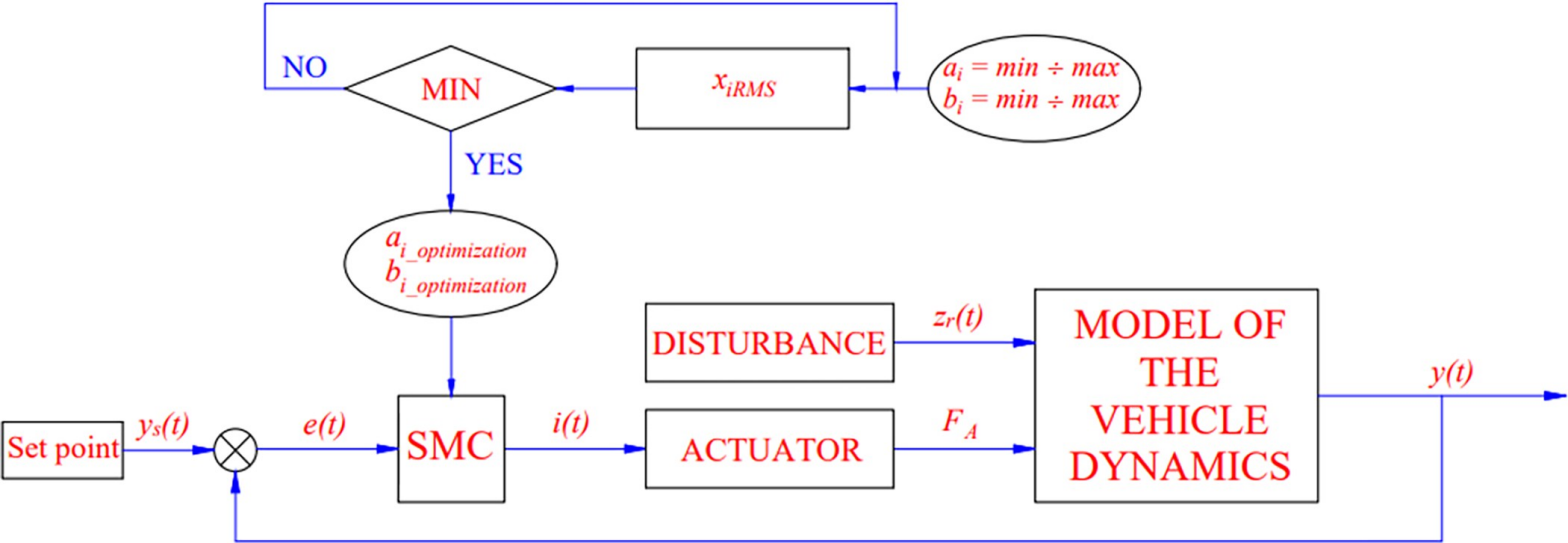
OSMC algorithm.

The in-loop optimization procedure is responsible for determining the values of the equation’s parameters, *a*_*i*_ and *p*_*i*_ (27). Their values will be utilized to discover the optimal value that reduces the number of oscillations experienced by the vehicle. The overall structure of the OSMC algorithm is presented below, and it may be seen in Fig 3.

This algorithm can be briefly described as follows:

**First step:** Defines the range of parameters. The optimal value to find will be in this range of values. The calculation will take a long time if this range is too large. Conversely, if this process range is too small, the accuracy will not be high. The selection of the value range of the parameters needs to satisfy the condition of the vehicle’s stability. The force generated by the actuator should not be too great because it can cause the wheel to lift off the road. Conversely, the impact force should not be too small. This range of values can be selected through many calculations and simulations.

**Second step:** Use an in-loop algorithm to run the a_i_ and b_i_ values from minimum to maximum. After each survey, the average value of the oscillation is calculated (RMS).

**Third step:** Evaluation of the average value just obtained from the survey cases. We can find the optimal coefficients of *a*_*i*_ and *b*_*i*_ with the minimum average value.

## 3. Results

### 3.1. Investigation cases

**Fig 4:**
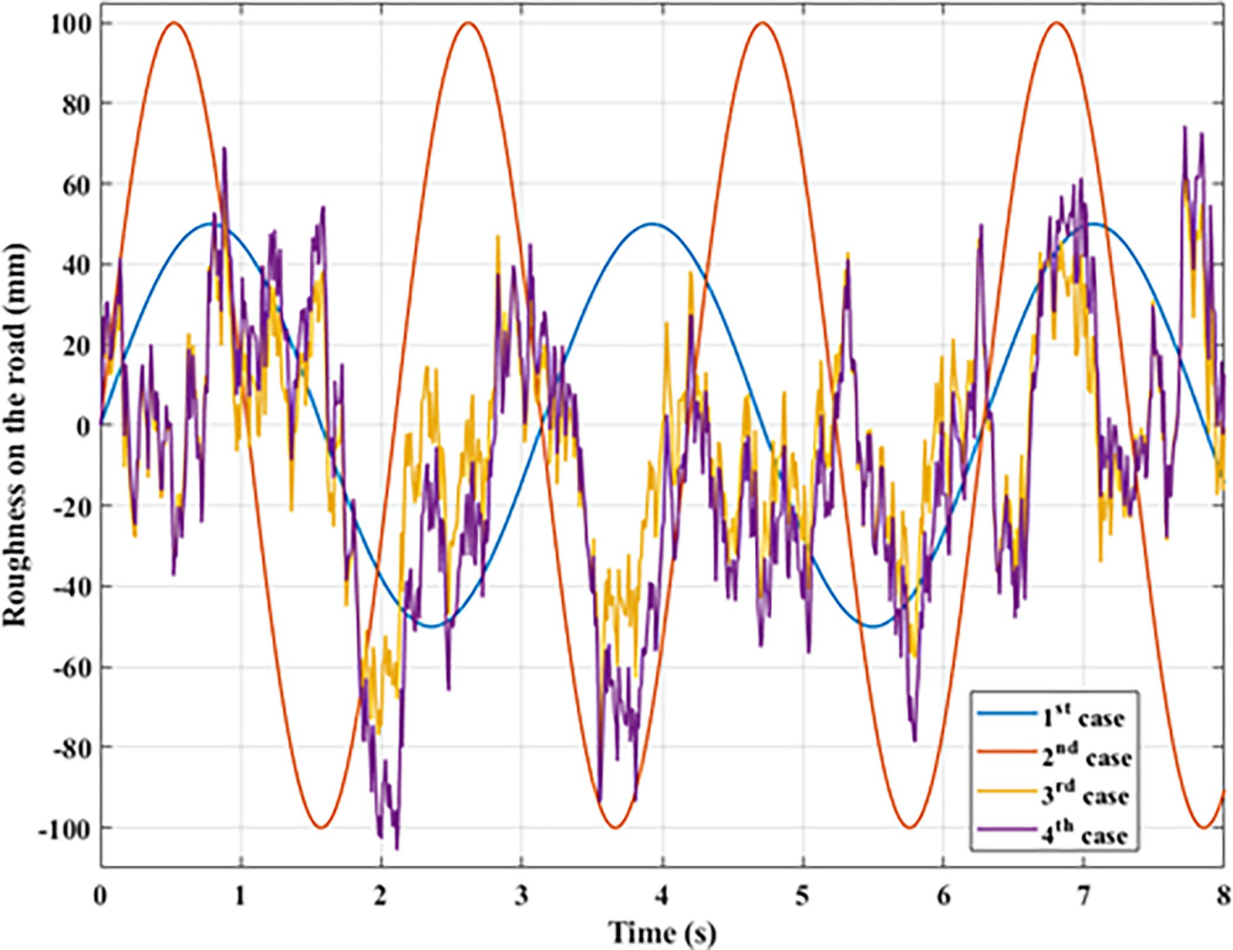
Roughness on the road.

In this investigation, only simulations were carried out. MATLAB-Simulink is the program that is responsible for running the simulation. When calculating the vibration of a vehicle, it is necessary to consider four distinct forms of excitations that come from the pavement. These four types of stimulation correlate to the four associated instances (Fig 4). Both the amplitude and the frequency of the oscillation are going to be altered as a result of the investigation instances. The numbers produced as a result of solving the issue include things like the dynamic load and the voltage produced by the controller. Other values include the displacement of the sprung mass and its acceleration. Their maximum and average values are considered.

The parameters used for the simulation are given in Table 1.

**Table 1:** Vehicle specification.

Symbol	Description	Value	Unit
m_s_	Sprung mass	560	kg
m_u_	Un-sprung mass	49	kg
C	Damper coefficient	3350	Nsm^-1^
K	Spring coefficient	41500	Nm^-1^
K_T_	Tire coefficient	180000	Nm^-1^
κ_1_	Actuator coefficient	539561	N^3/2^kg^-1/2^m^-1/2^V^-1^
κ_2_	Actuator coefficient	1	s^-1^
κ_3_	Actuator coefficient	5512500	Nm^-1^

### 3.2. Results and discussions

**Fig 5:**
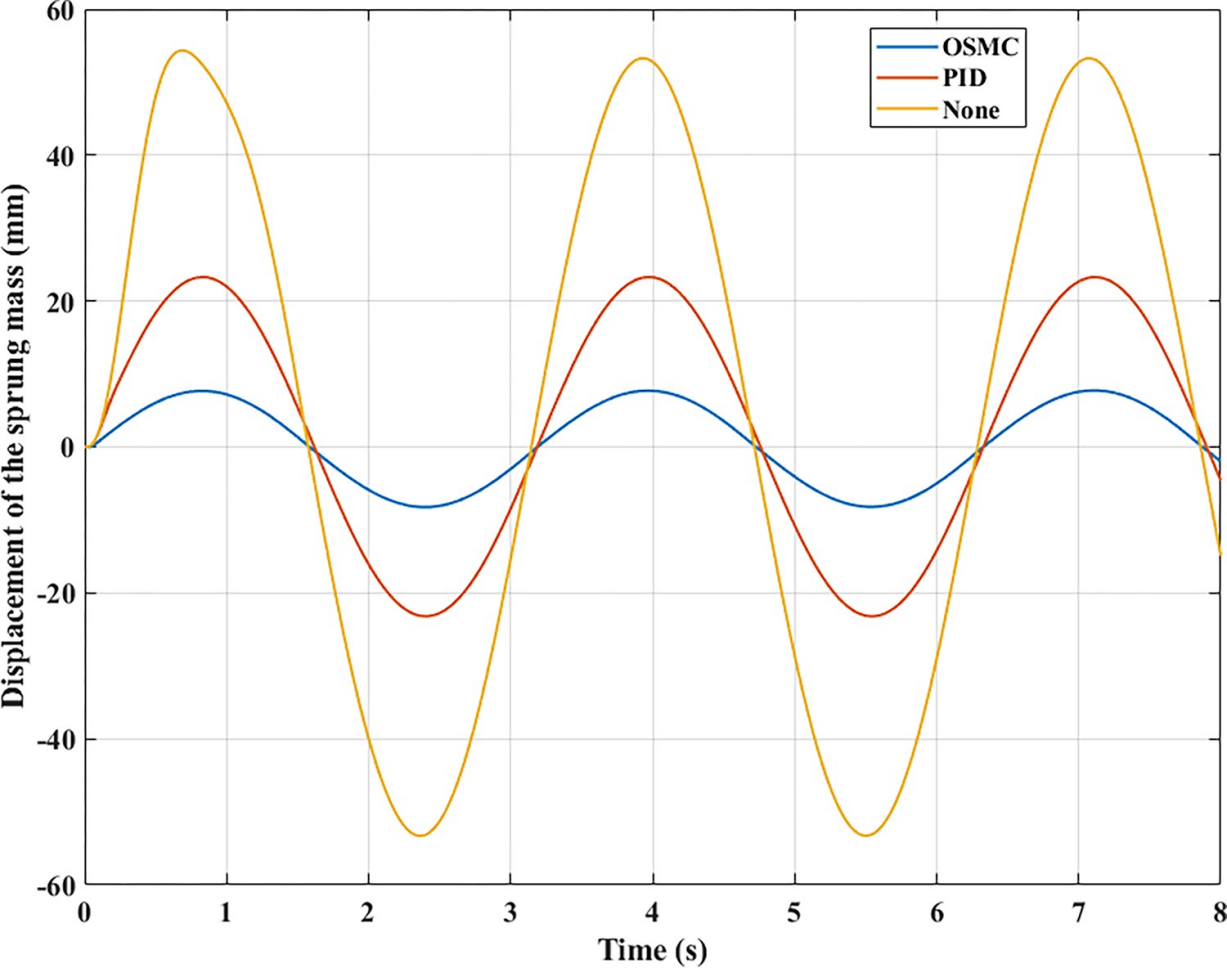
Displacement of the sprung mass (case 1).

**Fig 6:**
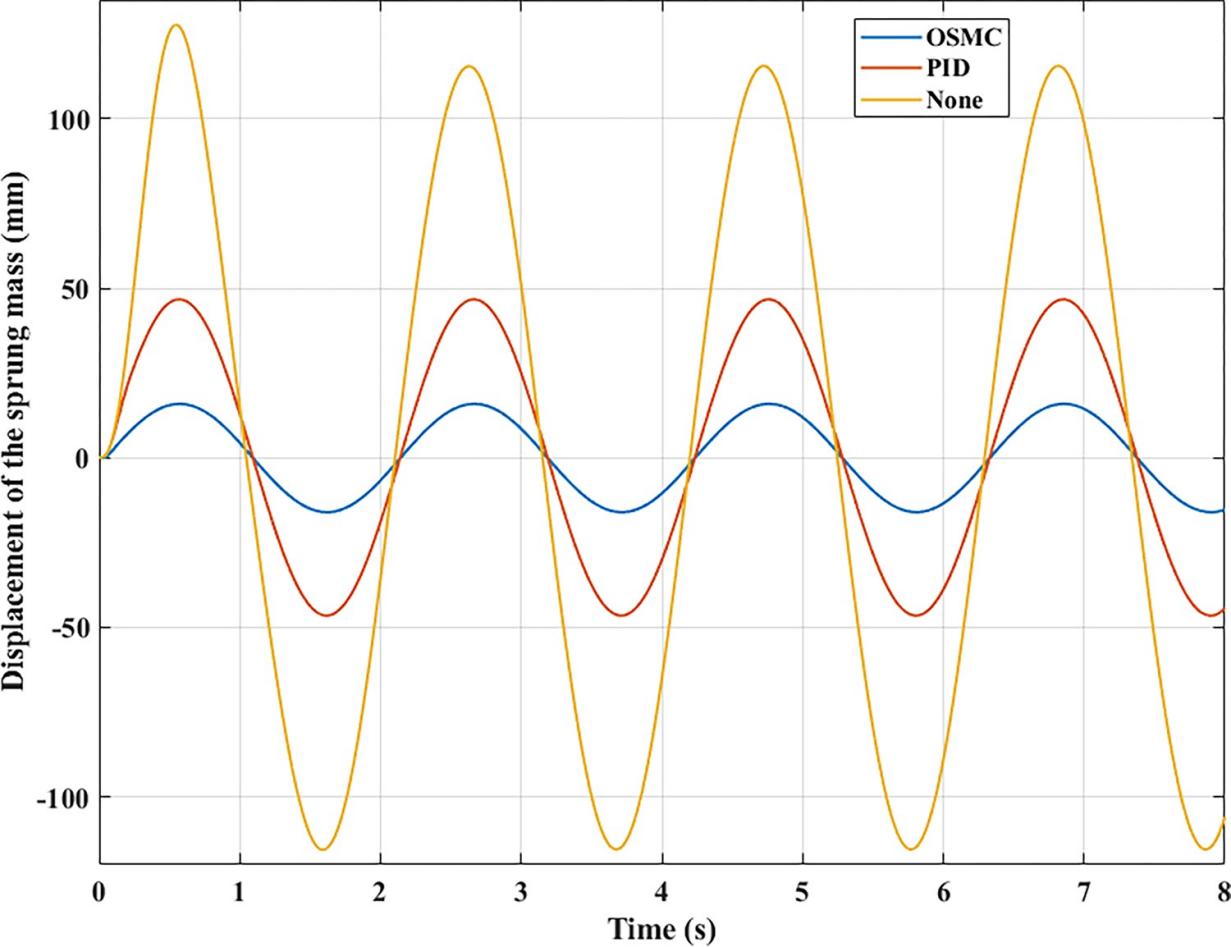
Displacement of the sprung mass (case 2).

**Fig 7:**
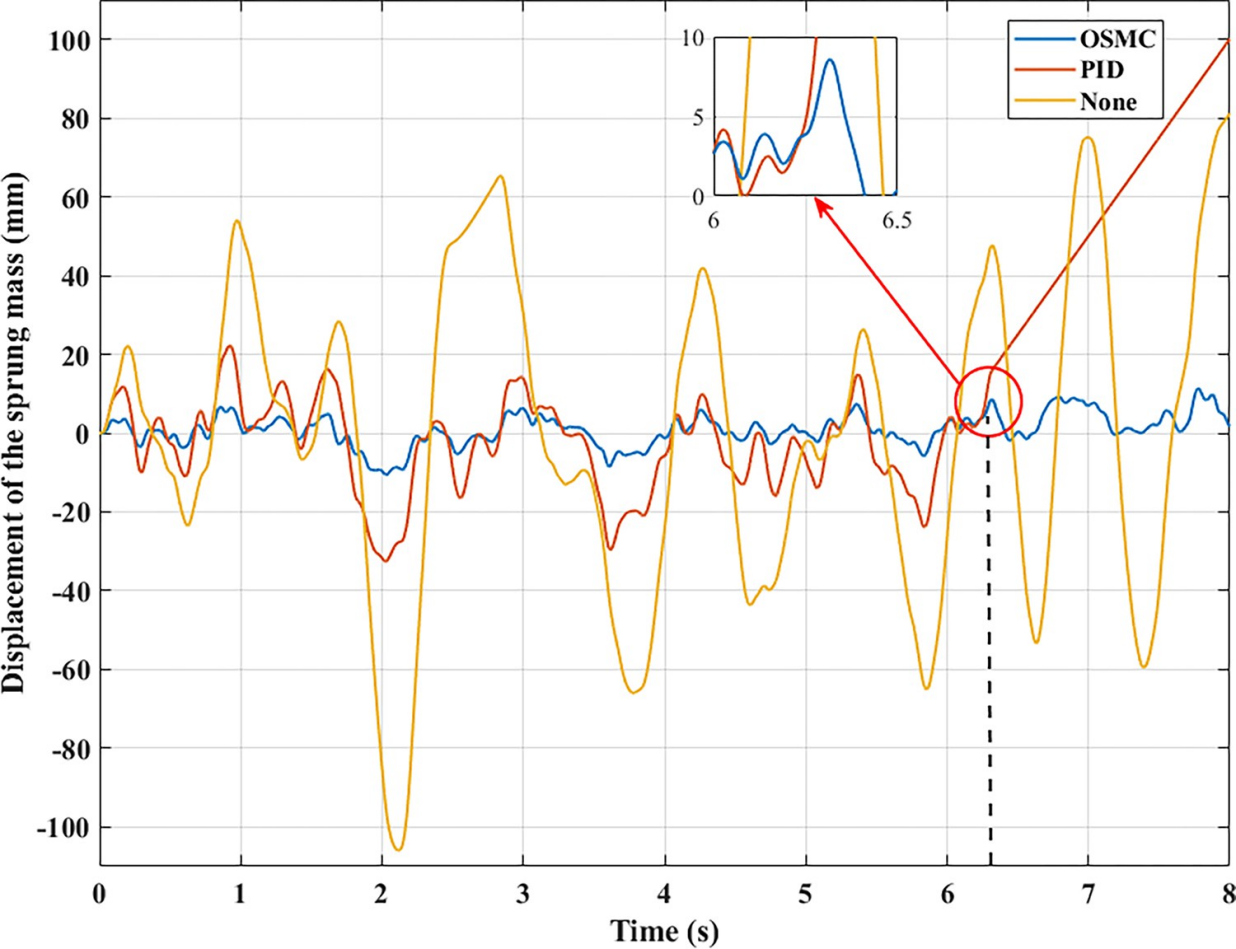
Displacement of the sprung mass (case 3).

**Fig 8:**
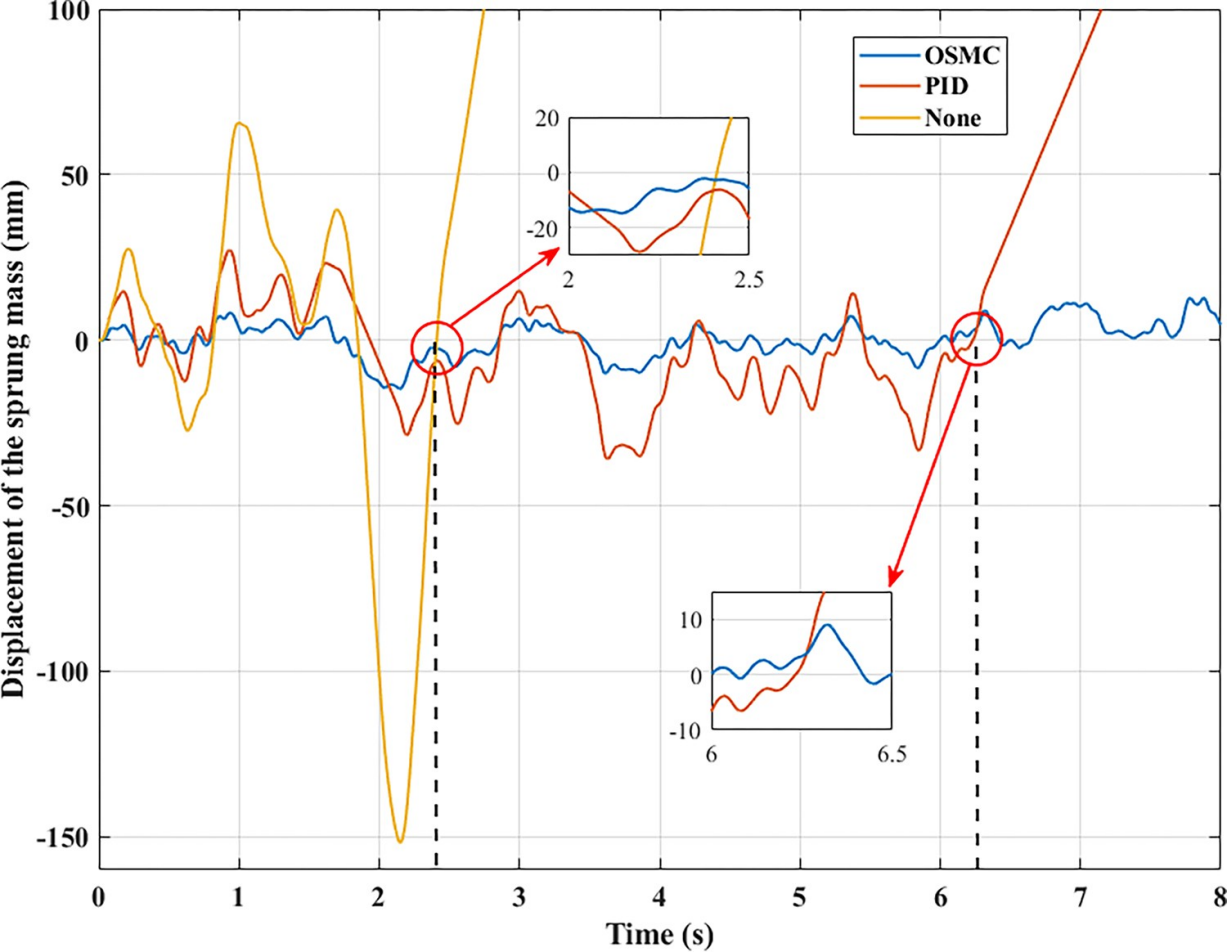
Displacement of the sprung mass (case 4).

The computation and simulation outcomes are depicted in the graphs below. Figs 5–8 illustrate the body displacement over time. According to this conclusion, the OSMC algorithm for the active suspension system minimizes the value of the displacement of the sprung mass. On the contrary, its value can be increased if only a mechanical suspension system is installed. In the first case (a), its highest value reaches 54.35 (mm), 23.29 (mm), and 8.23 (mm) in the three examination scenarios. In addition, the average values determined using the RMS criterion are 37.33 (mm), 16.29 (mm), and 5.54 (mm), respectively. In the second scenario (b), the oscillation’s amplitude and frequency have been enhanced.

Consequently, the vehicle body’s displacement values are likewise raised. The second example has maximum values of 127.68 (mm), 46.69 (mm), and 16.06 (mm), and mean values of 82.19 (mm), 33.07 (mm), and 11.31 (mm). These are merely cyclic excitations. Hence the value change is also cyclic.

In the remaining two instances, random stimulation was employed. Consequently, the oscillation of the vehicle will also change continually throughout time. In the third situation (c), if the car does not have an active suspension system, the body vibration can reach 105.97 (mm). In comparison, the maximum displacement value of the sprung mass is only approximately 11.34 (mm) when the vehicle has an active suspension system with a hydraulic actuator. This result validates using the OSMC algorithm to control the suspension system. If the PID algorithm is applied, the wheel has been lifted off the pavement at time t = 6.35 (s). In the last situation, vehicle instability exists (d). According to the results of Fig 5d, the wheel will be lifted off the road at a period of t = 2.49 (s) when the vehicle’s passive suspension system is utilized alone. In addition, instability may occur even if the vehicle has an active suspension system. Despite the application of the PID algorithm for the SISO system, this instability develops at time t = 6.34 (s). Meanwhile, the OSMC algorithm helps to maintain the vehicle’s performance stability during movement.

For random excitations, resonance can occur occasionally. This phenomenon occurs when the oscillation frequencies of the masses are equal, and it will cause the displacement of the vehicle body to reach its maximum value. This can cause instability and loss of comfort for users. In fact, the stiffness of the spring and the tire is different, so it is possible to limit the occurrence of resonance partially. Besides, under the action of hydraulic actuators, resonance phenomena can also be better prevented.

**Fig 9:**
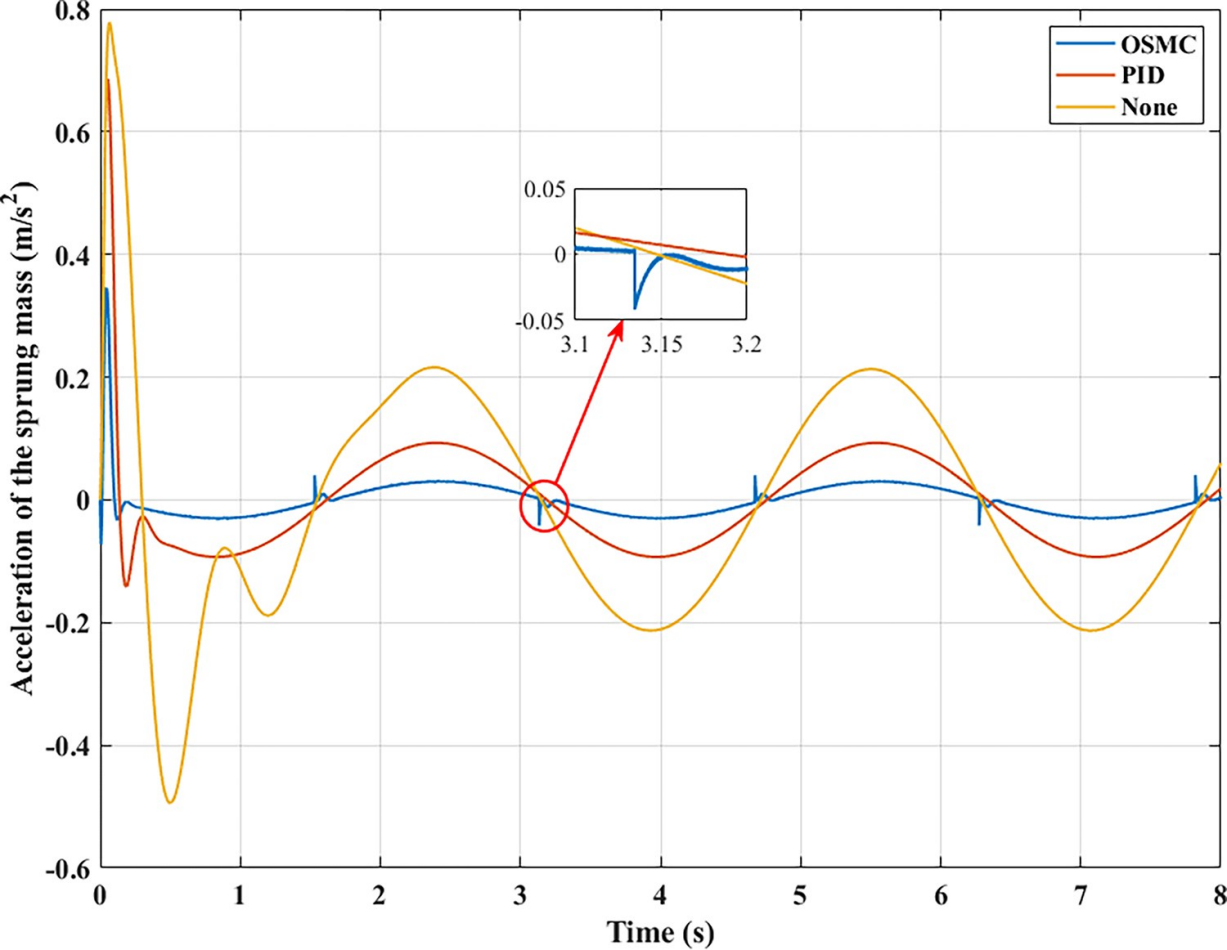
Acceleration of the sprung mass (case 1).

**Fig 10:**
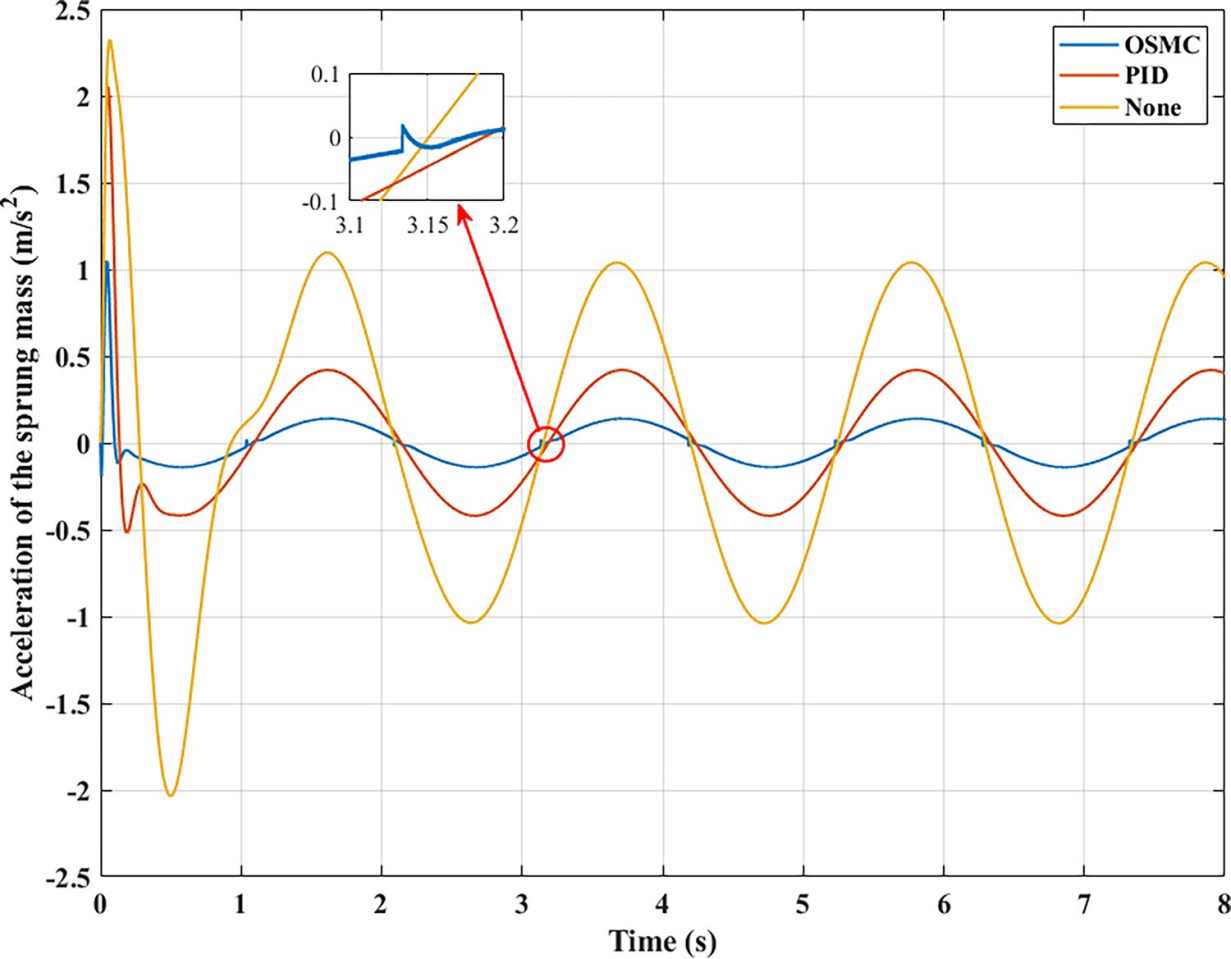
Acceleration of the sprung mass (case 2).

**Fig 11:**
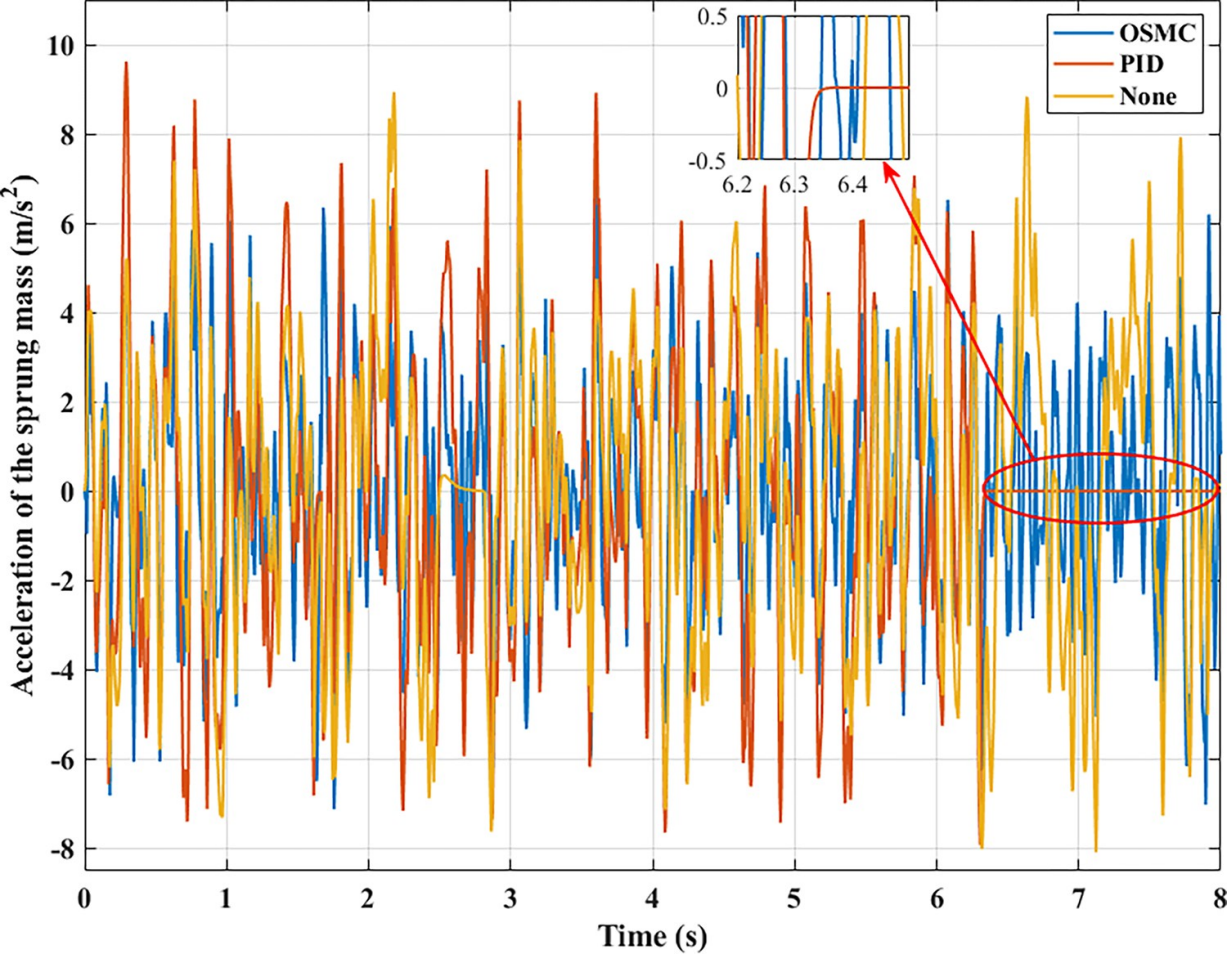
Acceleration of the sprung mass (case 3).

**Fig 12:**
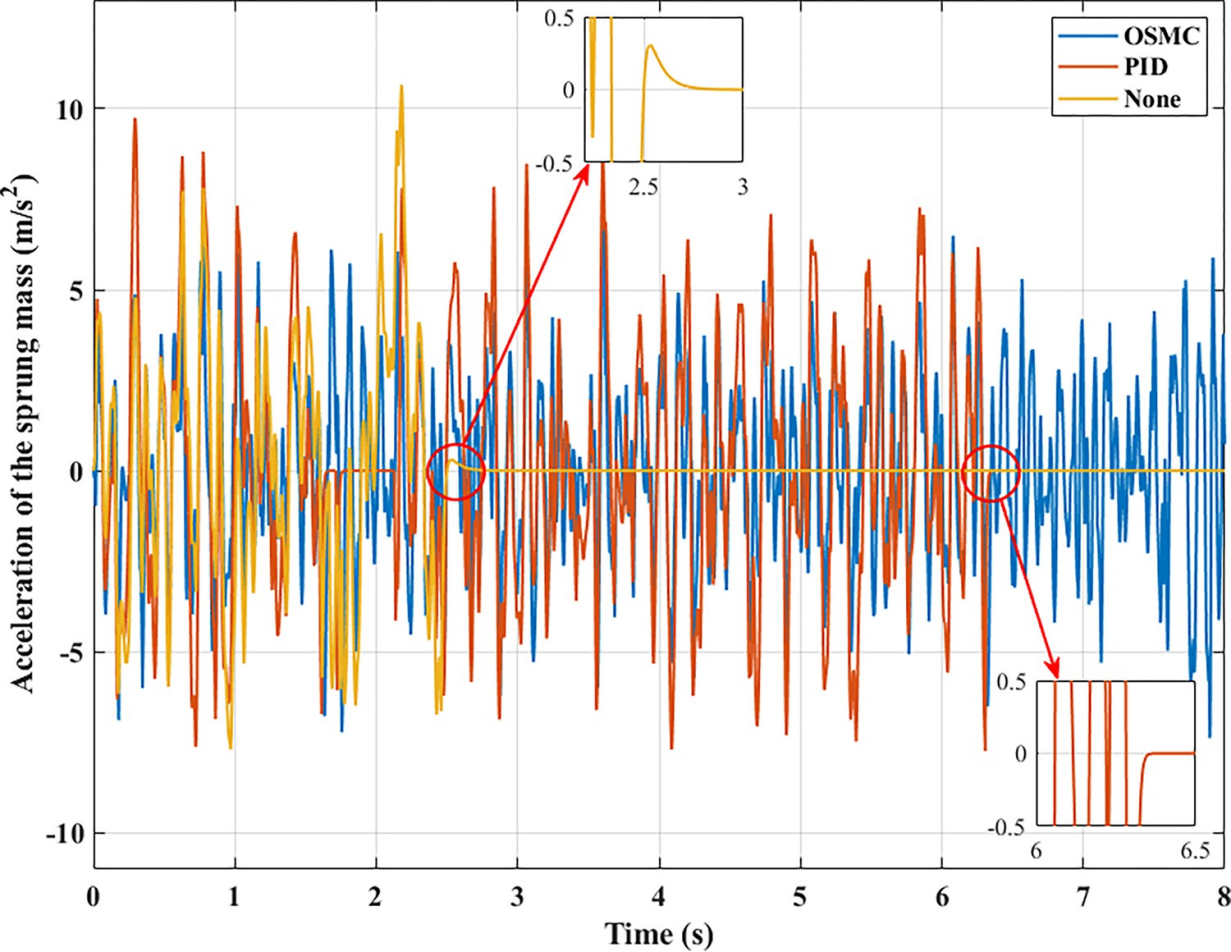
Acceleration of the sprung mass (case 4).

To evaluate the road’s smoothness, the acceleration of the sprung mass is taken into account. Figs 9–12 depict the change in acceleration of the sprung mass. In the first two instances, acceleration reaches its maximum value at the initial instant. For the first example, their maximum values correspond to the three analysed conditions as follows: 0.78 (m/s^2^), 0.69 (m/s^2^), and 0.34 (m/s^2^); 2.32 (m/s^2^), 2.05 (m/s^2^), and 1.05 (m/s^2^) for the second case. After the initial phase of the oscillation, the vehicle body’s acceleration will progressively decrease and frequently fluctuate over time. In the first scenario, their average values are 0.19 (m/s^2^), 0.09 (m/s^2^), and 0.03 (m/s^2^); in the second situation, they are 0.84 (m/s^2^), 0.35 (m/s^2^), and 0.12 (m/s^2^). In the latter two circumstances, random oscillation will cause the vehicle’s acceleration to vary more. In the third scenario (c), acceleration values vary continuously over time. Maximum and average acceleration values may reach 8.94 (m/s^2^) and 3.22 (m/s^2^) if the vehicle has a simple mechanical suspension. The wheel will be lifted off the road when a vehicle employs an active suspension system with a PID controller. From this moment, the vehicle body’s acceleration will decrease to zero. The vehicle body’s maximum acceleration previously achieved could be 9.62 (m/s^2^). This might increase the loss of the stability in the car. However, the ride comfort and stability can be enhanced by employing the OSMC algorithm for the suspension system. This improvement is negligible, 7.90 (m/s^2^) and 2.52 (m/s^2^). In the final scenario, the OSMC controller’s performance is completely used. When the nonlinear control technique is employed, the vehicle continues to oscillate in a stable manner, whereas wheel separation has occurred in the other two instances.

**Fig 13:**
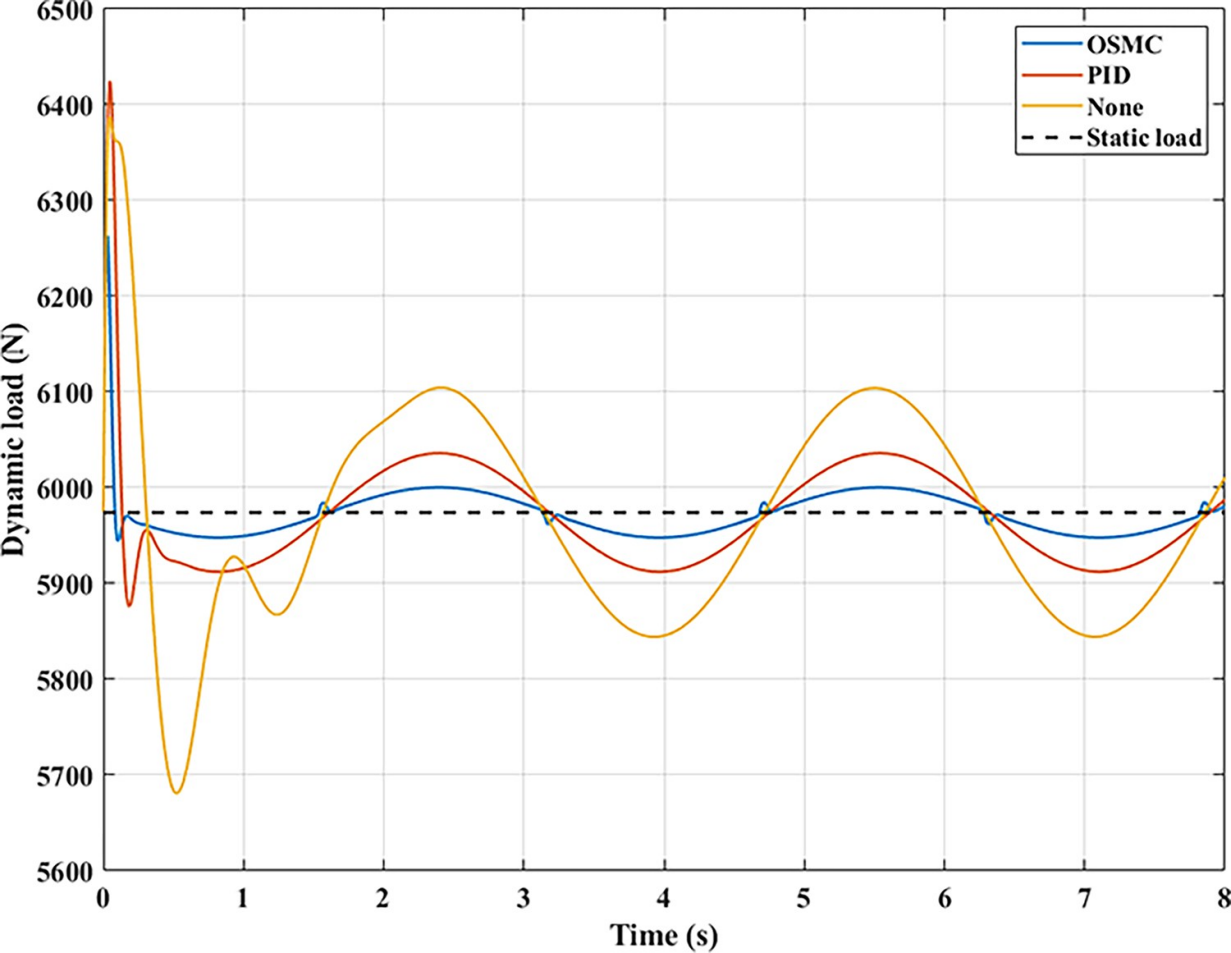
Dynamic load (case 1).

**Fig 14:**
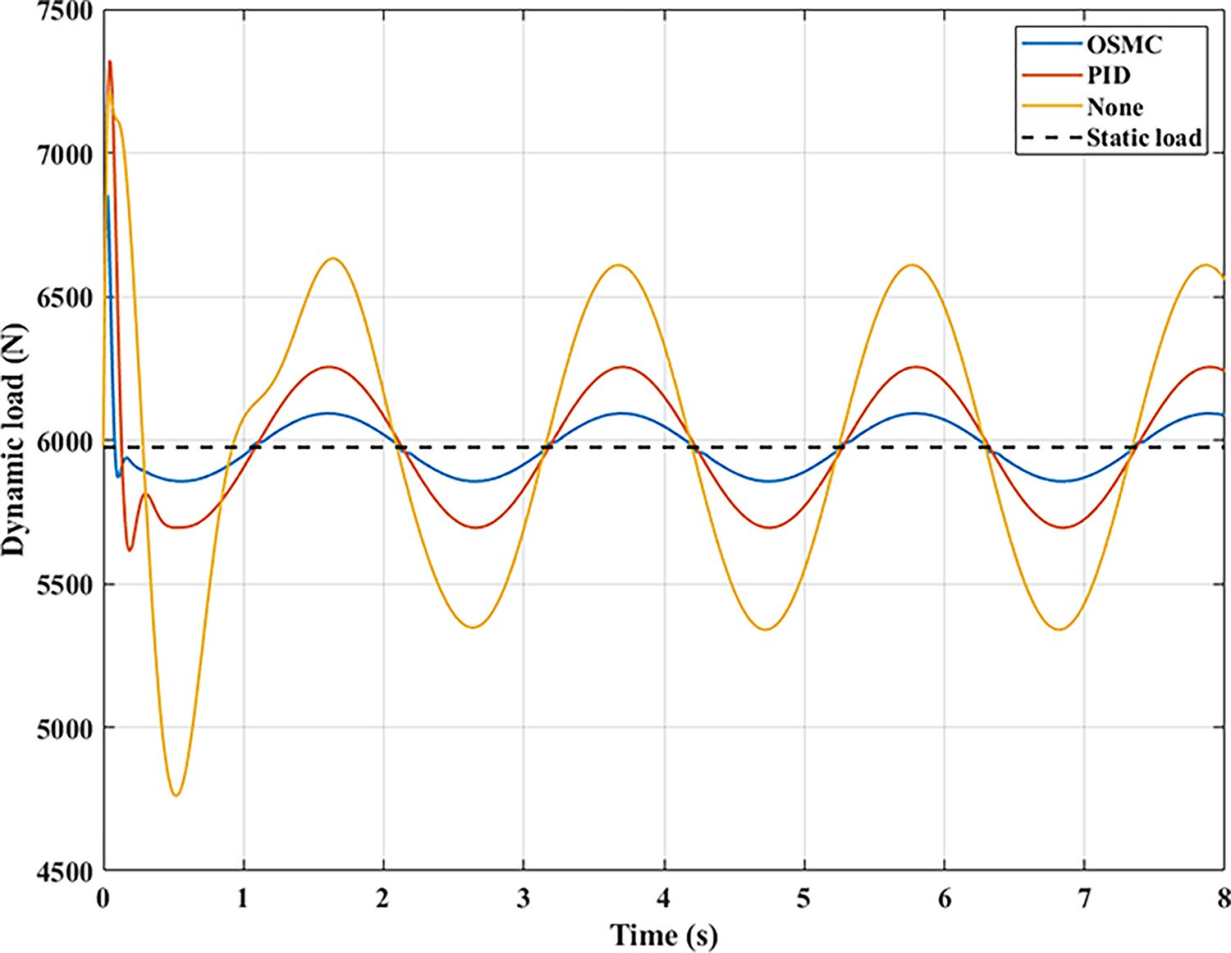
Dynamic load (case 2).

**Fig 15:**
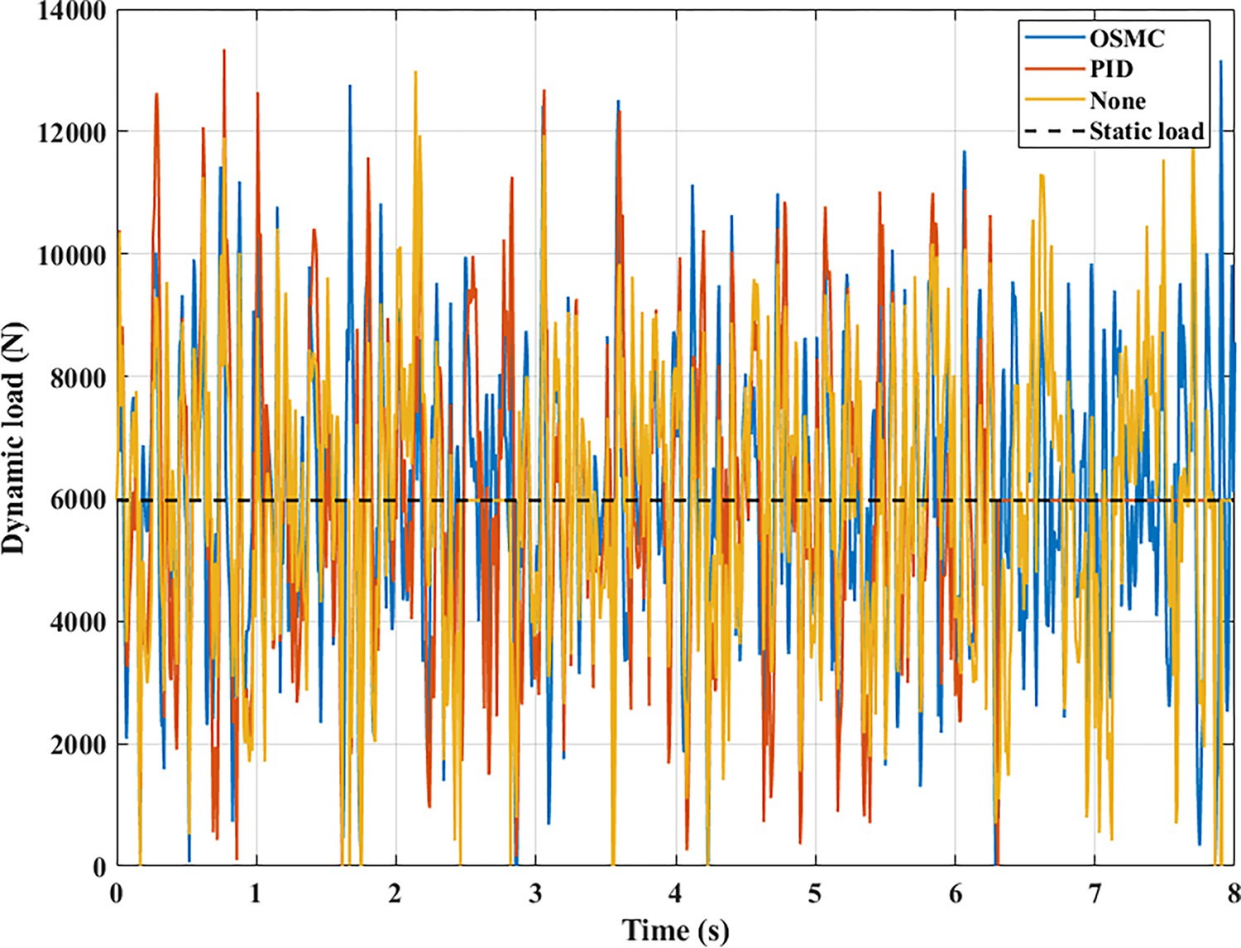
Dynamic load (case 3).

**Fig 16:**
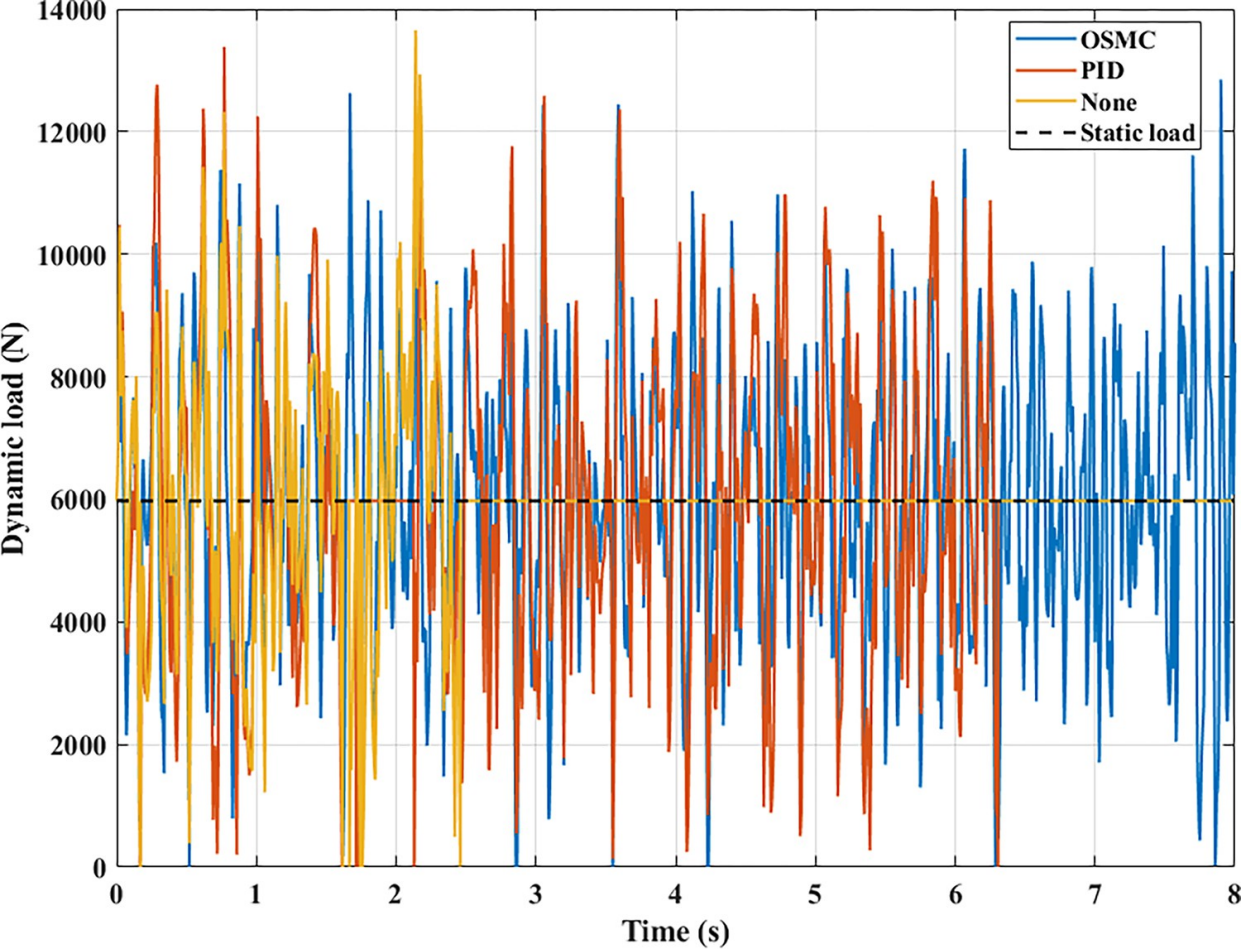
Dynamic load (case 4).

**Fig 17:**
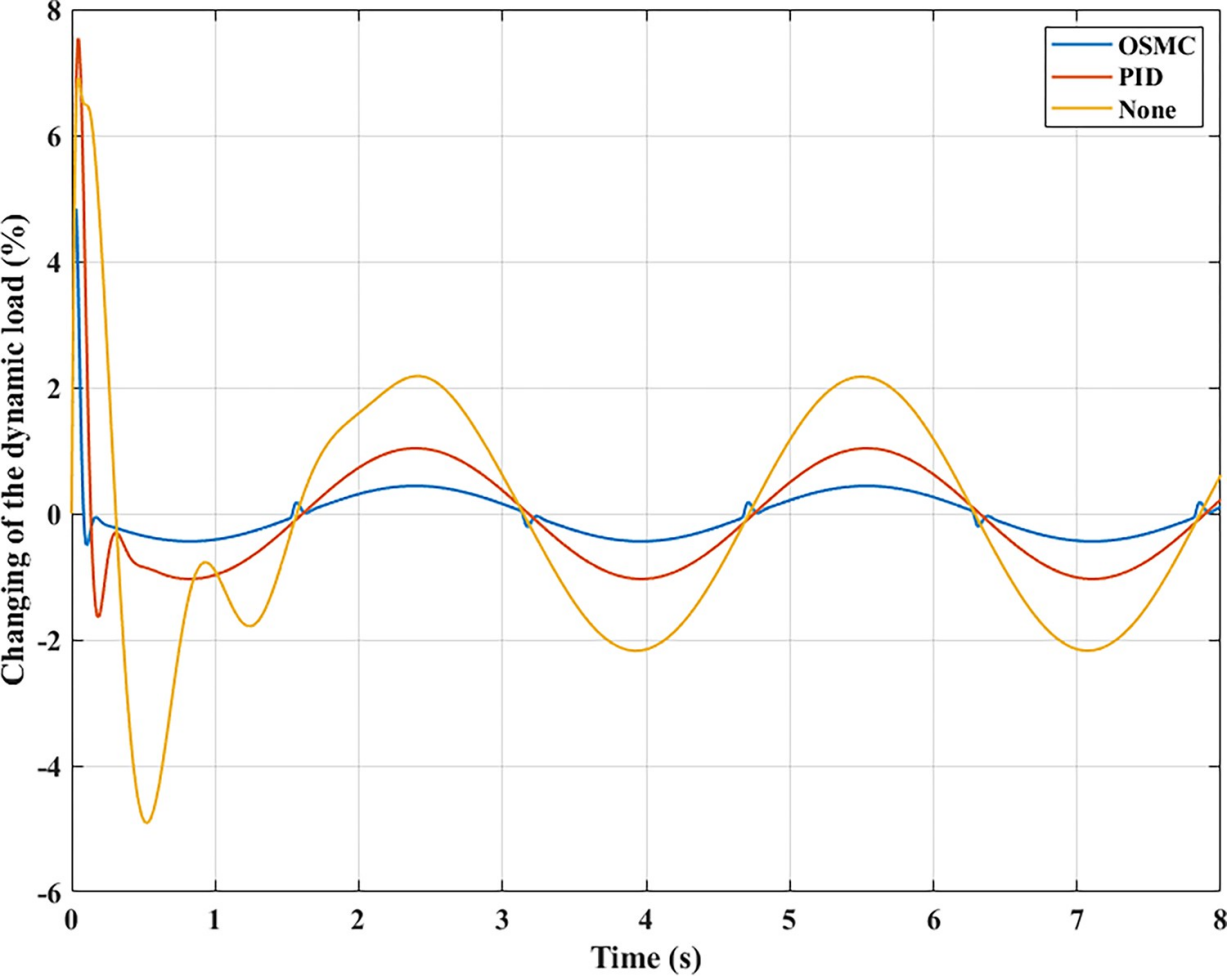
Changing the dynamic load (case 1).

**Fig 18:**
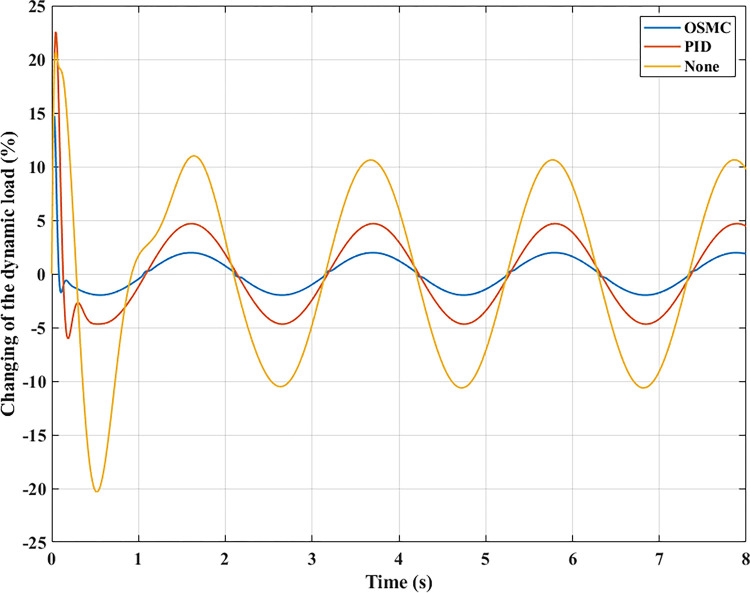
Changing the dynamic load (case 2).

**Fig 19:**
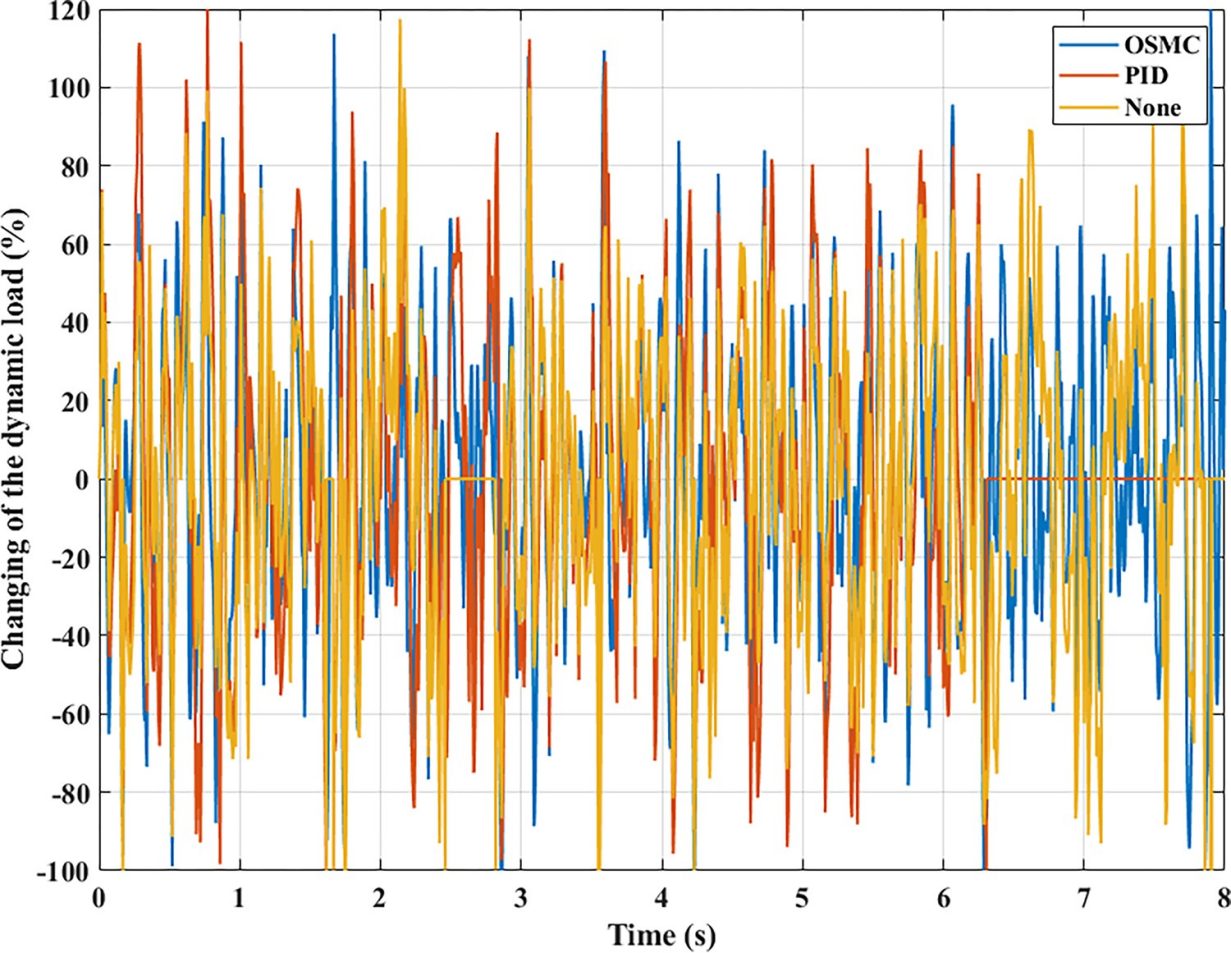
Changing the dynamic load (case 3).

**Fig 20:**
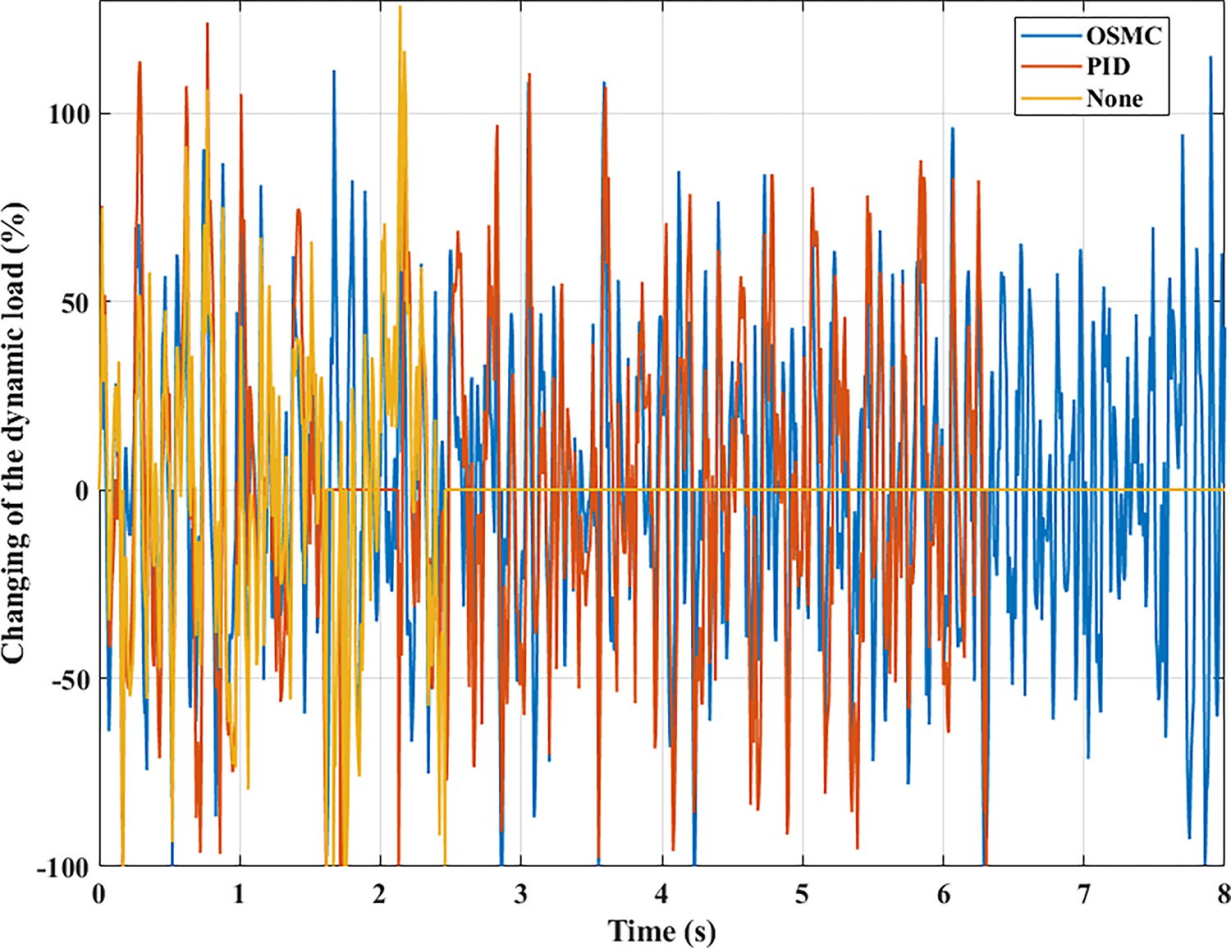
Changing the dynamic load (case 4).

The interaction between the wheel and the road surface is indicated by the changing wheel load or the dynamic load (Figs 13–16). With cyclic oscillation, the load variation is minimal (Figs 17–20), and the wheel remains in place. With random oscillations, the load variation is more pronounced. In rare instances, the dynamic load approaches zero, indicating that the wheel is separated from the road surface. At this point, there will be instability. If the wheel separation only lasts for a little time, the instability will be minimal. In contrast, if the load variation continues to increase, the wheels will be incapable of interacting with the road surface. Changes in dynamic load should not exceed -100% in order to maintain the vehicle’s stability on the road.

## 4. Conclusions

The roughness of the road surface is what causes car vibrations when driving. This oscillation has the potential to impact people and cargo. These oscillations are mitigated by the active suspension system. This article examines and applies the nonlinear control algorithm for active suspension systems. This study includes a quarter-dynamics model with a hydraulic actuator. In the MATLAB-Simulink environment, computations and simulations are carried out. There are four simulated scenarios that correlate to the four forms of pavement excitation. Three situations are considered in this example.

According to simulation results, the maximum and average values of the displacement and acceleration of the vehicle body are significant when the passive suspension system is used alone. Using an active suspension system with a PID algorithm can marginally improve the vehicle’s vibrations. Nonetheless, this improvement is modest. In rare instances, even the vehicle’s vibration is more potent. When the OSMC algorithm controls the active suspension system, oscillation is reduced. When this algorithm is applied to the simulation process, the preceding values reach a steady level. Moreover, the wheels engage consistently well with the road surface. The phenomenon of wheel separation can be restricted more drastically.

The OSMC algorithm is significantly more sophisticated than conventional control methods. The success of this controller is contingent upon the design of the sliding surface and the choice of state vectors. The "chattering" problem persists on the output signal of the controller. However, its effect is negligible. The OSMC algorithm can be combined with the Fuzzy and PID algorithms to enhance the controller’s performance and stability. This problem will continue in the subsequent article. In addition, this article only shows the results of the simulation process. During the simulation, some problems were overlooked. It is necessary to conduct experiments to evaluate the effectiveness of the controller. This content may be carried out in the near future.

## Abbreviations

F_A_: Force of the hydraulic actuator, N
F_C_: Force of the damper, N
F_K_: Force of the spring, N
F_KT_: Force of the tire, N
F_i_: Force of the inertia, N
z_r_: Roughness on the road, m
z_s_: Displacement of the sprung mass, m
z_u_: Displacement of the un-sprung mass, m
